# Predictors of insecticide-treated bed nets use among pregnant women in Sierra Leone: evidence from the 2019 Sierra Leone Demographic Health Survey

**DOI:** 10.1186/s12936-024-05018-6

**Published:** 2024-06-19

**Authors:** Augustus Osborne, Camilla Bangura

**Affiliations:** Department of Biological Sciences, School of Basic Sciences, Njala University, PMB, Freetown, Sierra Leone

**Keywords:** Insecticide-treated nets, Malaria, Pregnant women, Predictors, Sierra Leone

## Abstract

**Background:**

Malaria remains a significant public health threat in Sierra Leone, particularly for pregnant women and their unborn children. Infection during pregnancy can lead to severe consequences, including maternal anaemia, low birth weight, premature birth, and even death. Therefore, preventing malaria during pregnancy is crucial for improving maternal and child health outcomes. This study investigated the predictors of insecticide-treated bed net (ITN) use among pregnant women in Sierra Leone.

**Methods:**

The study analysed the 2019 Sierra Leone Demographic and Health Survey data (SLDHS). The study comprised a total of 900 pregnant women aged 15–49 years, representing the nationally representative sample**.** A multivariable binary regression analysis was used to explore the predictors of ITN use. The regression results were presented using an adjusted odds ratio (AOR) with 95% confidence intervals (CI).

**Results:**

The study found that the prevalence of ITN use among pregnant women was 64.2 [60.4, 67.9] in Sierra Leone. Pregnant women who were married [aOR = 2.02, 95% CI 1.32, 3.07] had higher odds of bed net use than those who were unmarried. Pregnant women with five or more children [aOR = 1.69, 95% CI 1.01, 2.84] had higher odds of mosquito bed net use than those with four and below children. Pregnant women living in the Northern, Northwestern, Southern and Western regions all had lower odds of bed net use than those in the Eastern region, with the lowest odds among those living in the western region [aOR = 0.19, 95% CI 0.09, 0.40]. Pregnant women who were Muslims [aOR = 0.63, 95% CI 0.41, 0.95] had lower odds of mosquito bed net use than Christians. Pregnant women with female household heads [aOR = 0.65, 95% CI 0.44, 0.95] had lower odds of mosquito bed net use than those with male household heads.

**Conclusion:**

ITN use among pregnant women in Sierra Leone remains suboptimal. Marital status, parity, sex of household head, region and religion were associated with bed net use. The government and policymakers in Sierra Leone should integrate ITN education and distribution into prenatal care services, emphasizing the benefits for both mother and baby—partnering with healthcare providers to raise awareness and encourage consistent use. Involve local leaders, religious figures, and mothers' groups to promote the benefits of ITN during pregnancy. Educate husbands and partners on the importance of ITN use during pregnancy and encourage their support in its consistent use.

## Background

Malaria, a vector-borne disease, is a significant worldwide health issue, particularly in sub-Saharan Africa, with pregnant women and children under five being the most severely impacted [1, 2]. In 2019, the World Health Organization (WHO) anticipated that 11 million pregnant women were exposed to malaria infections. West Africa had the highest prevalence of low-birth-weight infants related to malaria in pregnancy, resulting in 872,000 children being delivered by pregnant mothers with low birth weight [1]. Additionally, around 25 million pregnant women are currently vulnerable to contracting malaria. Malaria infection in pregnant women causes more than 10,000 maternal and 200,000 neonatal deaths per year [3, 4].

Sierra Leone is heavily affected by parasitic diseases, such as malaria, schistosomiasis, lymphatic filariasis, onchocerciasis, soil-transmitted helminth infections, and African trypanosomiasis [5, 6]. In 2019, the Global Burden of Disease Study reported that Sierra Leone had a malaria burden of 824,000 (355,000–1,400,000) disability-adjusted life years (DALYs) per 100,000 people and an onchocerciasis burden of 23,000 (9,750–40,000) DALYs per 100,000 inhabitants [7]. Sierra Leone recorded 2,615,850 cases of malaria in 2019, resulting in an incidence rate of 33.5%. Additionally, there were 6824 malaria-related fatalities, leading to a case fatality rate of 0.3%. Approximately 2.24 million outpatient visits annually were attributed to malaria, with around 1 million involving children under five years old [8]. Parasitic diseases, particularly malaria, have presented significant challenges to the health and economic progress of Sierra Leone.

Malaria during pregnancy is linked to severe illness and death for both the mother and the child due to adverse maternal health and birth complications such as miscarriage, stillbirth, and intrauterine growth retardation [9, 10]. The WHO suggests utilizing ITN, Intermittent Preventive Therapy in pregnancy (IPTp), early diagnosis, and efficient treatment to prevent and manage malaria during pregnancy [11]. Using ITNs is advantageous for preventing malaria in regions with high transmission rates and is, therefore, advised together with other preventive measures [12]. Due to the high probability of reduced immunity in pregnant women, it is crucial to use ITN even in regions with low disease transmission rates [9]. The goals for malaria interventions during pregnancy, particularly the timely use of ITN and IPTp, have often not been achieved despite remaining problems that need to be addressed [13, 14].

Current malaria policy and interventions for pregnant women in Sierra Leone include intermittent preventive treatment with sulfadoxine-pyrimethamine (IPTp-SP) and ITN distribution on their first ante-natal care visit [8]. This is the cornerstone of malaria prevention for pregnant women in Sierra Leone. Insecticide-treated bed nets (ITNs) are also universally distributed through campaigns and encouraged for regular use, especially during pregnancy and early childhood [15]. Targeted social and behaviour change communication (SBCC) campaigns emphasize the importance of preventive measures like ITNs and IPTp-SP to increase awareness and understanding. Rapid diagnostic tests (RDTs) are also used to accurately diagnose malaria at all healthcare system levels, ensuring prompt and appropriate treatment. Pregnant women with suspected or confirmed malaria receive a prompt diagnosis, treatment, and counselling on prevention and adherence to IPTp-SP [8, 15]. Despite these improvements in the country, rural communities often face distance and resource limitations, hindering consistent preventive care and treatment. Also, not all pregnant women receive all recommended doses due to various factors, including limited access to care or misconceptions about the medication. Challenges like ITN damage, discomfort, or lack of proper hanging practices reduce their effectiveness [8, 15]. Continued efforts are crucial to address the challenges, improve policy implementation, and increase access to effective preventive and treatment interventions for pregnant women in Sierra Leone.

Several studies have explored the sociodemographic and behavioural factors influencing bed net use, including household size, number of nets available, education level, and awareness of benefits [16–25]. However, limited research has examined the specific factors influencing the adoption of ITNs among pregnant women in Sierra Leone**.** Understanding these factors is crucial to improving the country's maternal and child health outcomes.

Previous studies [26–31] on malaria in Sierra Leone have focused on the testing, diagnosis, management, and treatment of malaria and no study has looked at the factors associated with ITN use among pregnant women who are particularly vulnerable. Therefore, this study aims to identify the specific factors that hinder ITN use among pregnant women in Sierra Leone using the 2019 SLDHS. Understanding these factors is crucial to improving the country's maternal and child health outcomes. By identifying the factors of ITN use in this population, this study can inform targeted interventions and policy improvements to promote ITN use and optimize malaria prevention for pregnant women in Sierra Leone.

## Methods

### Data source and design

The 2019 SLDHS data was used for this study [32]. SLDHS was conducted over four months (from May 2019 to August 2019) to gather data on demographic, health, and nutritional factors among women, children, and men [33]. A cross-sectional design was adopted for the SLDHS, and respondents were sampled using a multistage sampling method. In the first stage, 578 enumeration areas (EAs), consisting of 214 urban and 364 rural regions, were selected. A systematic selection procedure was employed for the second stage to select 24 households from each EA. This selection process ultimately resulted in a sample size of 13,872 households. A detailed description of the survey methodology is available in the literature [33].

This study included 900 pregnant women aged 15–49 with complete datasets on the variables of interest in the SLDHS. The dataset was accessed following the procedures outlined on the official DHS program website [32]. The study was done in accordance with the Strengthening Reporting of Observational Studies in Epidemiology (STROBE) guidelines [34].

### Study variables

#### Outcome variable

This study utilizes a binary measure of ITN use derived from the SLDHS question asking women if they slept under an ITN the night before the survey (Yes/No). Previous research [16] used a coding scheme (Yes = 1, No = 0) for this question.

#### Explanatory variables

Sixteen explanatory variables were included in the study. The variables include the age of the women, place of residence, level of education, wealth index, employment status, region, parity, marital status, sex of household head, exposure to media (newspapers/magazines, radio, and television), religion, distance to the health facility, visited a health facility in the last 12 months, and visited by a fieldworker in the last 12 months. These variables were selected based on their statistically significant association with ITN use from previous studies [16, 21, 22, 25] and their availability in the DHS data. Table 1 shows the categories of the variables included in the study.

**Table 1:** Background characteristics of the pregnant women in Sierra Leone (n = 900)

Variables	Weighted frequency (n)	Weighted percentage (%)
Age (years)		
15–24	390	41.2
25–34	434	42.6
35–49	166	16.3
Region		
Eastern	204	21.2
Northern	228	20.9
Northwestern	195	20.8
Southern	261	22.8
Western	102	14.3
Type of place of residence		
Urban	292	32.2
Rural	698	67.8
Education level		
No education	546	51.9
Primary	145	16.0
Secondary	280	29.7
Higher	19	2.4
Religion		
Christian	197	20.7
Islam	793	79.3
Sex of household head		
Male	796	79.5
Female	194	20.5
Wealth index		
Poorest	267	24.9
Poorer	236	23.0
Middle	198	19.9
Richer	165	17.1
Richest	124	15.1
Parity		
Below four	838	85.0
Five and above	152	15.0
Pregnancy trimester		
First	204	21.5
Second	402	40.3
Third	384	38.2
Visited by fieldworker in last 12 months		
No	686	67.1
Yes	304	32.9
Visited health facility in last 12 months		
No	203	21.2
Yes	787	78.8
Distance to a health facility		
Big problem	522	50.3
Not a big problem	468	49.7
Covered by health insurance		
No	954	96.0
Yes	36	4.0
Marital status		
Unmarried	210	23.2
Married	780	76.8
Currently working		
No	274	28.1
Yes	716	71.9
Exposure to media		
No	552	52.6
Yes	438	47.4

### Data analysis

The data was analysed using SPSS version 28. The complex sampling command on SPSS for weighting and complex sampling design was used. Percentage and confidence intervals (CI) were used to present the prevalence of mosquito bed net use among pregnant women and their distribution across the explanatory variables. A chi-square test of independence was conducted to determine the variables significantly associated with the unmet need for contraception at p < 0.05. The variance inflation factor (VIF) was used to test for evidence of collinearity among the variables studied. The results showed that the highest and lowest VIF were 2.43 and 1.03. Hence, there was no evidence of high collinearity among the variables. Later, binary logistic regression analysis was performed to examine the variables associated with ITN use among pregnant women. The results were presented using adjusted odds ratio (AOR) with their respective 95% confidence interval (CI). Statistical significance was set at p < 0.05.

## Results

### Background characteristics of the pregnant women in Sierra Leone

Table 1 shows the background characteristics of the 900 pregnant women included in the study, most between 25 and 34 years old (42.6%). Southern (22.8%) and Northern (20.9%) are the most represented regions. Most of the pregnant women live in rural areas (67.8%). The majority of the pregnant women have no education (51.9%). Most of the pregnant women are Muslim (79.3%). The household head is male in most cases (79.5%). The poorest and poorer wealth index categories represent almost half of the pregnant women (47.9%). Most pregnant women have had fewer than four children (85.0%). The pregnant women are almost evenly distributed across the three trimesters of pregnancy. About one-third of the pregnant women have been visited by a field worker in the last 12 months (32.9%). Most pregnant women have visited a health facility in the last 12 months (78.8%). Distance to the health facility is a big problem for half of the pregnant women (50.3%). Only a tiny percentage of pregnant women are covered by health insurance (4.0%). The majority of the pregnant women are married (76.8%). Most of the pregnant women are currently working (71.9%). Almost half of the pregnant women have no exposure to media (52.6%).

### Prevalence and distribution of ITN use among pregnant women in Sierra Leone

Table 2 shows the bivariate analysis of mosquito bed net use among pregnant women in Sierra Leone, using a Pearson chi-square test to compare the proportions of ITN use across different categories of variables. The overall prevalence of ITN use among pregnant women was 64.2%. ITN use was significantly associated with age, region, residence, sex of household head, wealth index, parity, a visit to a health facility in the last 12 months, and marital status with p-values less than 0.05. ITN use was not significantly associated with education level, religion, exposure to media, distance to health facility, pregnancy trimester, and visit by fieldworker in the last 12 months, with p-values greater than 0.05.

**Table 2:** Bivariate analysis of ITN uses among pregnant women in Sierra Leone

Variables	ITN use	P-value
	Yes % [CI]	
Prevalence of ITN use	64.2 [60.4, 67.9]	
Age (years)		< 0.001
15–24	35.9 [31.9, 40.1]	
25–34	46.0 [41.7, 50.4]	
35–49	18.0 [14.9, 21.6]	
Region		< 0.001
Eastern	25.2 [21.6, 29.3]	
Northern	21.6 [17.8, 25.9]	
Northwestern	20.4 [16.8, 24.5]	
Southern	24.4 [20.3, 29.0]	
Western	8.5 [6.3, 11.3]	
Residence		0.007
Urban	28.3 [24.3, 32.7]	
Rural	71.7 [67.3, 75.7]	
Education level		0.394
No education	54.2 [49.1, 59.2]	
Primary	16.0 [13.2, 19.4]	
Secondary	27.6 [23.2, 32.5]	
Higher	2.1 [1.1, 4.0]	
Religion		0.469
Christian	21.6 [17.4, 26.4]	
Islam	78.4 [73.6, 82.6]	
Sex of household head		< 0.001
Male	82.8 [79.4, 85.8]	
Female	17.2 [14.2, 20.6]	
Wealth index		0.003
Poorest	26.4 [22.7, 30.5]	
Poorer	23.9 [20.4, 27.7]	
Middle	20.8 [17.2, 24.9]	
Richer	17.8 [14.4, 21.8]	
Richest	11.2 [8.6, 14.3]	
Parity		< 0.001
Below four	81.7 [78.3, 84.6]	
Five and above	18.3 [15.4, 21.7]	
Pregnancy trimester		0.424
First	20.5 [17.2, 24.2]	
Second	39.6 [35.9, 43.5]	
Third	39.8 [36.1, 43.7]	
Visited by fieldworker in last 12 months		0.733
No	66.7 [62.2, 70.9]	
Yes	33.3 [29.1, 37.8]	
Visited health facility in last 12 months		0.023
No	18.5 [15.2, 22.3]	
Yes	81.5 [77.7, 84.8]	
Distance to a health facility		0.693
Big problem	50.9 [45.8, 56.1]	
Not a big problem	49.1 [43.9, 54.2]	
Covered by health insurance		0.643
No	96.3 [94.1, 97.7]	
Yes	3.7 [2.3, 5.9]	
Marital status		< 0.001
Unmarried	18.0 [14.1, 22.8]	
Married	82.0 [77.2, 85.9]	
Currently working		0.658
No	27.5 [23.5, 31.8]	
Yes	72.5 [68.2, 76.5]	
Exposure to media		0.444
No	53.6 [49.1, 58.1]	
Yes	46.4 [41.9, 50.9]	

^*^P-values are generated from the Chi-Square test

### Predictors associated with the use of ITN among pregnant women in Sierra Leone

Table 3 shows the predictors associated with ITN use among pregnant women in Sierra Leone. Pregnant women who were married [aOR = 2.02, 95% CI 1.32, 3.07] had higher odds of bed net use than their unmarried counterparts. Pregnant women with five or more children [aOR = 1.69, 95% CI 1.01, 2.84] had higher odds of ITN use than those with four or fewer children. Pregnant women living in the Northern, Northwestern, Southern and Western regions all had lower odds of ITN use than those living in the Eastern region, with the lowest odds among those living in the Western region [aOR = 0.19, 95% CI 0.09, 0.40]. Pregnant women who were Muslims [aOR = 0.63, 95% CI 0.41, 0.95] had lower odds of ITN use than Christians. Pregnant women who had females as household heads [aOR = 0.65, 95% CI 0.44, 0.95] had lower odds of ITN use than males.

**Table 3:** predictors associated with the use of ITN among pregnant women in Sierra Leone

Variables	Mosquito bed nets use
Age (years)	aOR [95% CI]
15–24	Ref.
25–34	1.37 [0.97, 1.92]
35–49	1.27 [0.74, 2.18]
Region	
Eastern	Ref.
Northern	0.62*** [0.34, 1.10]
Northwestern	0.57*** [0.33, 0.99]
Southern	0.74*** [0.45, 1.20]
Western	0.19*** [0.09, 0.40]
Type of place of residence	
Urban	Ref.
Rural	0.89 [0.47, 1.69]
Education level	
No education	Ref.
Primary	1.08 [0.68, 1.70]
Secondary	1.09 [0.69, 1.70]
Higher	1.10 [0.30, 3.97]
Religion	
Christian	Ref.
Islam	0.63** [0.41, 0.95]
Sex of household head	
Male	Ref.
Female	0.65** [0.44, 0.95]
Wealth index	
Poorest	0.99 [0.44, 2.19]
Poorer	0.98 [0.45, 2.12]
Middle	1.09 [0.52, 2.26]
Richer	1.55 [0.86, 2.79]
Richest	Ref.
Parity	
Below four	Ref.
Five and above	1.69** [1.01, 2.84]
Pregnancy trimester	
First	Ref.
Second	1.03 [0.68, 1.54]
Third	1.08 [0.71, 1.64]
Visited by fieldworker in last 12 months	
No	Ref.
Yes	1.05 [0.75, 1.46]
Visited health facility in last 12 months	
No	Ref.
Yes	1.39 [0.91, 2.13]
Distance to a health facility	
Big problem	0.81 [0.55, 1.17]
Not a big problem	Ref.
Covered by health insurance	
No	Ref.
Yes	0.67 [0.31, 1.44]
Marital status	
Unmarried	Ref.
Married	2.02*** [1.32, 3.07]
Currently working	
No	Ref.
Yes	0.77 [0.51, 1.17]
Exposure to media	
No	Ref.
Yes	1.10 [0.77, 1.55]

aOR: adjusted odds ratio; 95% CI: 95% Confidence Interval; ref: reference category

** p < ; 0.01, *** p < ; 0.001

## Discussion

This study examined the predictors of ITN use among pregnant women in Sierra Leone. The results showed that 64% of the pregnant women used mosquito bed nets. Region, religion, sex of household head, parity, and marital status were the predictors associated with pregnant women's ITN use in Sierra Leone. This finding is higher than the 61% in Ghana [20], 57% reported in Rwanda [16], 39% in Ethiopia [22] and 35% in Uganda [19]. Findings are, however, lower than the 71% reported in the Democratic Republic of Congo [35]. The potential reason behind the sub-optimal 64% rate among pregnant women in Sierra Leone may be due to unequal access to bed nets in certain regions, which can hinder their availability. Access to ITNs might be lower in remote rural areas than urban centres, leaving pregnant women in these regions more vulnerable. A lack of understanding about the effectiveness of bed nets in preventing malaria can deter their use [36]. Cultural or religious beliefs conflicting with bed net use, as observed with lower use among Muslims, can act as barriers [36]. Certain cultural practices might prioritize traditional medicine or rituals over modern medical interventions like ITNs. Concerns about heat, stuffiness, or difficulty sleeping under bed nets can be deterrents [37, 38]. Further research, including qualitative studies and intervention evaluations, is needed to promote more equitable ITN use across Sierra Leone. This will ultimately contribute to a nationwide reduction in malaria burden among pregnant women and improve maternal and child health outcomes in Sierra Leone.

Married pregnant women had higher odds of ITN use than those who were unmarried. This finding is consistent with the previous study in Rwanda [16]. Spouses might provide financial support for purchasing or maintaining bed nets, participate in hanging and treating them, and encourage their use [39]. Spouses might have better access to information or healthcare services, increasing their knowledge about malaria prevention and the benefits of bed nets. There was also a higher likelihood of ITN use among pregnant women with five or more children in Sierra Leone compared to those with four or fewer. Larger families might be more likely to share bed nets, though potentially impacting individual protection [39].

Pregnant women living in the Northern, Northwestern, Southern, and Western regions had lower odds of ITN use, and pregnant women in Sierra Leone's Western region showed significantly lower odds of using bed nets than those in the Eastern region. The findings from the study are contrary to the findings reported in Rwanda [16]. Variations in ITN distribution programmes across regions could lead to disparities in access. Regions with less robust distribution networks might have fewer ITNs readily available [36]. Regional economic disparities could impact affordability. Pregnant women in poorer regions might struggle to purchase ITNs even if they are not freely distributed [37]. Rural areas in the Eastern region might have better access to healthcare facilities and providers who actively promote bed net use compared to urban areas with potentially overburdened healthcare systems [40]. By understanding the reasons behind this regional disparity, targeted interventions and tailored promotion strategies can be developed to address pregnant women's specific needs and challenges in the Western region, ultimately improving bed net use and contributing to malaria prevention efforts in Sierra Leone.

The finding that pregnant Muslim women had lower odds of ITN use than Christians is consistent with the previous study in Uganda [41]. It is crucial to acknowledge the complex and sensitive nature of this topic and that attributing lower bed net use solely to religion can be misleading and oversimplifying. In-depth qualitative studies and community engagement are essential to understanding the reasons behind this observed association and identifying culturally appropriate solutions. Understanding the nuances beyond just religious affiliation can help to develop targeted interventions and communication strategies that address the specific needs and challenges faced by various communities in Sierra Leone, ultimately leading to improved bed net use and malaria prevention for all. This study also found a link between female household heads and lower bed net use among pregnant women in Sierra Leone. The reasons behind this association are complex and require further investigation.

### Policy and practice implications

The study on predictors of ITN use among pregnant women in Sierra Leone offers valuable insights for policymakers and programme implementers. The government and policymakers should develop culturally sensitive messaging tailored to specific regions, religious beliefs, and the needs of women in female-headed households. Utilize diverse communication channels (radio, mobile health, community forums) featuring trusted local voices and addressing regional variations in beliefs and practices. Collaborate with religious leaders to develop messages that resonate with their communities and address misconceptions about ITNs. Explore public–private partnerships to encourage private sector involvement in ITN distribution and sales. Consider cost-recovery mechanisms alongside free or subsidized ITN programmes, ensuring affordability for low-income households—partner with local leaders, influencers, and healthcare workers to promote ITN use within communities. However, funding constraints might limit the reach and effectiveness of SBCC campaigns and access programmes. Navigating religious beliefs and cultural norms requires careful planning and collaboration with community leaders. Strategies to overcome these barriers include seeking partnerships with international donors, the private sector, and NGOs to secure long-term funding for SBCC campaigns and ITN access programmes. Collaborate with existing local networks and healthcare infrastructure to optimize resource utilization and campaign reach. By employing these targeted SBCC campaigns that address regional variations, religious beliefs, and the needs of specific demographics, alongside efforts to improve ITN access and community mobilization, policymakers and programme implementers can significantly increase ITN use among pregnant women in Sierra Leone. This will contribute to a national reduction in malaria burden and improved maternal and child health outcomes.

### Strengths and limitations

One of the study's strengths is that the SLDHS provides data representative of the entire Sierra Leonean population, allowing for generalizable conclusions about pregnant women's bed net use across the country. Also, the SLDHS collects data on a wide range of sociodemographic, economic, and health-related factors, allowing for the exploration of various potential predictors of bed net use. The study, however, has some limitations. The SLDHS is a cross-sectional survey, which means it cannot establish causal relationships between factors and ITN use. The data relies on self-reported information, which may be subject to recall and social desirability biases. Multistage sampling can introduce selection bias if households within chosen enumeration areas (EAs) weren't randomly selected, and households with missing data or those that refused to participate could skew the results. The survey collects limited information on specific reasons for ITN use, compliance, or challenges faced. The limitations highlight the need for further research to strengthen the understanding factors influencing ITN use among pregnant women in Sierra Leone.

## Conclusion

ITN net use among pregnant women in Sierra Leone remains suboptimal (64.2%). Being married and having five or more children were associated with increased bed net use, possibly due to heightened awareness of malaria risk for themselves and their children. Certain regions, particularly the Western region, show significantly lower bed net use than the Eastern region. This highlights the need for regional-specific interventions. Pregnant Muslim women have lower odds of using bed nets compared to Christians. Culturally sensitive interventions addressing beliefs and practices might be needed. Having a female head of household was associated with lower bed net use. Further research is required to understand the underlying reasons. Government and policymakers in Sierra Leone should address the specific needs and concerns of the different groups, particularly in regions with low use. They should ensure readily available and affordable treated bed nets through distribution programmes or subsidies. They should also encourage community ownership and promote bed net use through local leaders and influencers. They should also collaborate with religious leaders and communities to develop culturally sensitive interventions that align with their beliefs and practices.

## Data Availability

Data is publicly available via the measure DHS website at https://dhsprogram.com/data/available-datasets.cfm.

## Abbreviations

AOR: Adjusted odds ratio
CI: Confidence interval
EA: Enumeration areas
ITN: Insecticide-treated nets
SLDHS: Sierra Leone Demographic and Health Survey
SSA: Sub-Saharan Africa
